# Joint effect of docosahexaenoic acid intake and tobacco smoke exposure on learning disability in children and adolescents: a cross-sectional study from the NHANES database

**DOI:** 10.1186/s13052-024-01745-3

**Published:** 2024-09-27

**Authors:** Ling Liu, Xiuli Shu, Zijun Xu, Haibo Jiang

**Affiliations:** 1 Department of Emergency, The Affiliated Hospital of Hangzhou Normal University, No. 126 Wenzhou Road, Gongshu District, Hangzhou, 310015 Zhejiang Province China; 2 College of Preschool Education, Nanyang Vocational College of Agriculture, Nanyang, 473000 Henan Province China

**Keywords:** Docosahexaenoic acid, Tobacco smoke exposure, Child learning disabilities, NHANES database

## Abstract

**Background:**

Docosahexaenoic acid (DHA) has been reported to be associated with the children’s neurodevelopment, who may be exposed to tobacco smoke simultaneously. The evidence about joint effect of DHA intake and tobacco smoke exposure on children and adolescents’ learning disabilities (LD) was limited. The objective of this study was to assess the joint effect of DHA intake and tobacco smoke exposure on children and adolescents’ LD.

**Methods:**

A cross-sectional analysis of the NHANES 1999–2004 was performed. Children and adolescents aged 6–15 years old were included. The outcome was diagnosed by parental report of ever health professionals or school representative-identified LD. Dietary DHA intake data were obtained by food frequency questionnaire and tobacco smoke exposure levels were evaluated by serum cotinine levels. Weighted univariable and multivariate logistic regression analyses were conducted to determine the joint effect of DHA intake and tobacco smoke exposure on LD in children and adolescents, with odds ratios (ORs) and 95% confidence intervals (CIs). This joint association was further assessed after stratification by age, gender, body mass index, the history of attention deficit disorder and seen mental health professional.

**Results:**

We identified 5,247 children and adolescents in present study, of whom 593 (11.30%) had LD. After adjusting covariates, we observed children and adolescents with DHA intake (OR = 0.76, 95%CI: 0.61–0.96) was related to lower incidence of LD; children who exposure to tobacco smoke was related to higher incidence of LD (OR = 1.54, 95%CI: 1.07–2.23); children and adolescents who exposure to tobacco smoke and without DHA intake were related to highest odds of LD (OR = 2.08, 95%CI: 1.37–3.17, *P* for trend = 0.042), that was, DHA and tobacco smoke exposure may have a joint effect on the odds of LD in children and adolescents. Subgroup analyses suggested this joint effect was robust especially among children and adolescents with normal & underweight BMI and without the history of attention deficit disorder and seen mental health professional.

**Conclusion:**

Increasing the DHA intake and reducing tobacco smoke exposure may have a potential role in the prevention of LD in children and adolescents. This joint effect warrants further investigation by large-scale prospective study.

**Supplementary Information:**

The online version contains supplementary material available at 10.1186/s13052-024-01745-3.

## Introduction

Learning disabilities (LD), a heterogeneous group of disorders, is featured by “persistent difficulties in reading, writing, arithmetic or mathematical reasoning skills during formal school years of schooling [1]. LD usually leads to academic achievement lower than individual’s intellectual capacity which they expected to obtain. In addition to significant academic problems, they are also accompanied by emotional/social/social adjustment problems, which seriously affect the normal psychological development of children and adolescents [2]. Based on nationally representative data, the estimated LD prevalence was 8.3% among children and adolescents aged 6–17 years old [3]. At present, a etiology including genetic, neuropathological and environmental factors has been proposed for LD in children and adolescents [4, 5].

Dietary behavior has been shown to be associated with children’s cognitive performance [6]. Docosahexaenoic acid (DHA) is a kind of long chain polyunsaturated fatty acid (PUFA) that needs to be supplied by the human diet and plays a vital role in the optimal development, maturation, and aging of neural structures. Available evidence suggest that increased erythrocyte DHA levels may improve behavior, attention and literacy in attention deficit hyperactivity disorder (ADHD) children [7, 8]. Tobacco smoke exposure as an important environment factor has been proven to be associated with a higher risk of a variety of neurological diseases including cognitive decline and schizophrenia, which may be centered on inflammatory and oxidative stress pathways [9, 10]. Noteworthily, individuals with inadequate DHA intake are equally likely to be exposed to tobacco smoke. Previous study has shown that there is an association between tobacco smoke exposure and reduced levels of omega-3 PUFA both in human and mice [11]. Moreover, Moore et al. [12, 13] reported that low level of omega-3 of PUFA may worsen the effects of exposure to smoke on obesity of children.

However, whether DHA intake and tobacco smoke exposure have a joint effect LD among children and adolescents remains unclear. Based on previous related studies, we speculate that children with DHA intake and non-tobacco smoke exposure would have a lower risk of LD. Hence, we evaluate the association between of DHA intake and tobacco smoke exposure and their joint effect on children and adolescents’ LD based on the National Health and Nutrition Examination Surveys (NHANES) 1999–2004.

## Methods

### Study design and participants

A cross-sectional analysis of the NHANES 1999–2004 was performed. NHANES is conducted biennially by the National Centers for Health Statistics (NCHS), the Centers for Disease Control and Prevention (CDC). It aims to evaluate the health nutritional status for a representative population of the U.S. population using the complex, multistage, probability sampling methods. The NHANES is a public survey and is granted by the NCHS Ethics Review Board, and all participants provided written informed consent. Therefore, no external ethic approval is required for this study.

### Study population

**Fig. 1:**
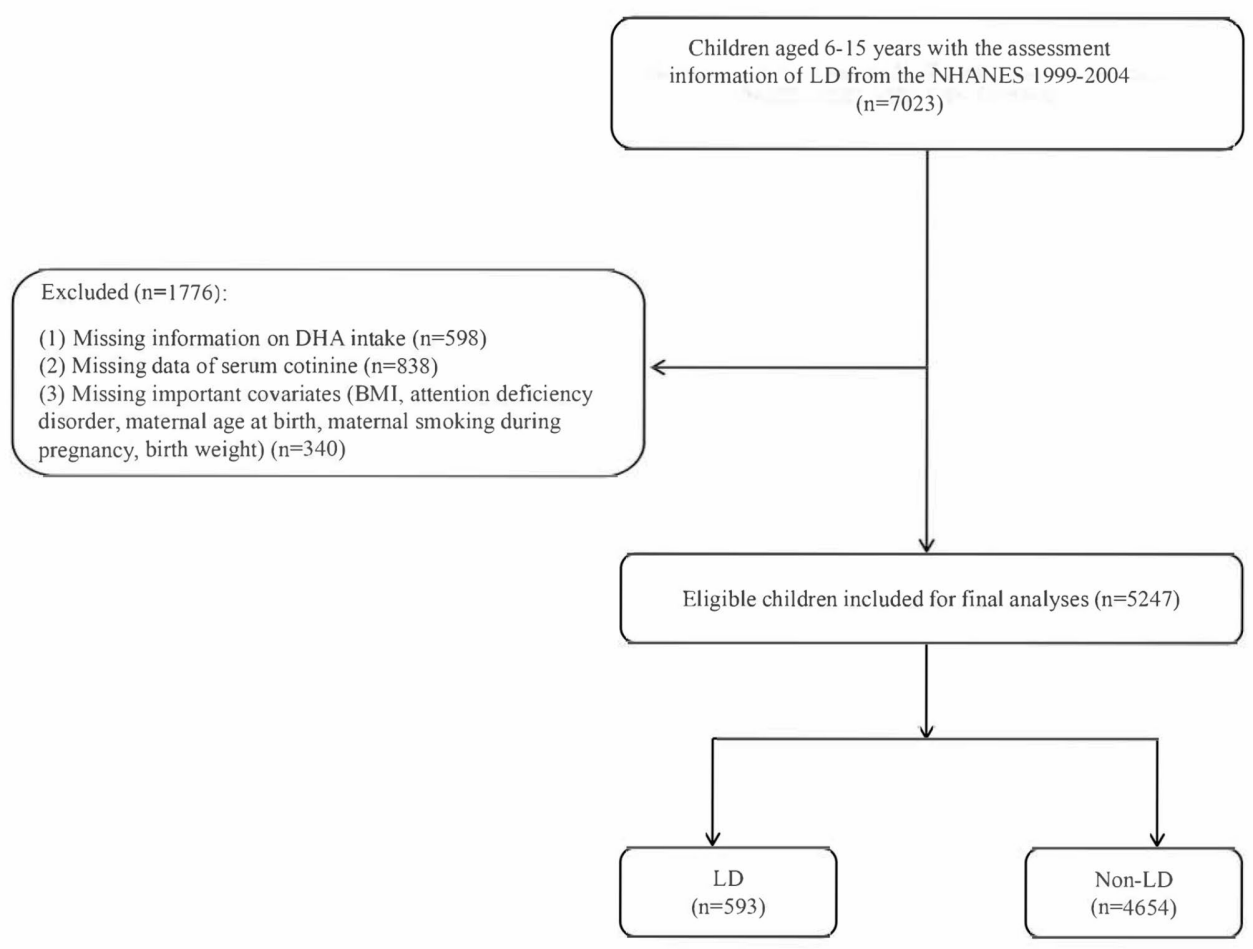
The flow chart of population screening

In present study, a total of 7,035 children and adolescents were initially included. Among these participants, 598 missing DHA intake information, 838 missing serum cotinine measurement information, 12 missing LD diagnostic information, 36 missing BMI data, 9 missing ADHD assessment information, 78 missing information of maternal age at delivery, 38 missing information of whether maternal smoking during pregnancy and 179 missing birth weight data were excluded. Finally, 5,247 eligible children and adolescents were included for further analysis. Figure 1 represents the flow chart of population screening.

### LD diagnosis

Information about the LD in children and adolescents was assessed by the parents or guardian’s response to the questionnaire “Has a representative from a school or a health professional ever told you that your child had a LD?” [14].

### DHA intake assessment

The dietary information of previous month was collected using a validated, table-based, DHA-specific, semiquantitative food frequency questionnaire (FFQ), named the DHA Intake Evaluation Tool [15]. FFQ included 75 types of seafood, 38 types of freshwater food and mutton. DHA-related supplements were also taken into account. A standard portion size was specified for each types of food, and participants were asked the frequency they had consumed that portion on average in the previous month. Consumption frequency contains 12 options, which ranged from “once per week” to “three times per day”, and 9 food intake options varied from 25 g to 250 g of edible portion.

### Tobacco smoke exposure assessment

Tobacco smoke exposure was defined as if a participants reported at least 1 smoker in their home or had a serum cotinine level more than 0.05 µg/L. Active smoke was assessed by answering the question “During the past 5 days, including today, did you smoke cigarettes, pipes, cigars, little cigars and cigarillos, water pipes or e-cigarettes?” positivity [16]. Isotope dilution high-performance liquid chromatography and atmospheric pressure chemical ionization tandem mass spectrometry were used to measure the serum cotinine levels. This method has good accuracy, with mean values within 9% of theoretical values at all levels expect the lower limit of quantification, where it was within 14% of the theoretical values. All samples were analyzed at the Division of Laboratory Sciences, National Center for Environmental Health, Centers for Disease Control and Prevention in Atlanta, Georgia. In present study, serum cotinine level ≥ 0.05 ng/mL were considered as tobacco smoke exposure, and < 0.05 ng/mL were considered as non-tobacco smoke exposure.

### Covariates

Demographic information and participants’ diseases history were extracted from the database. Gender, age and race were self-reported demographic information. Poverty-to-income ratio (PIR) was calculated as the ratio of the midpoint of the observed family income category to the family’s appropriate poverty threshold set by the US Census Bureau in a given calendar year [17]. PIR < 1 was defined as poor, PIR 1-1.83 was defined as near poor, 1.85–3.5 was defined as middle income and PIR > 3.5 was defined as higher income. Education level was assessed by the question “What is the highest grade or level of school you have completed or the highest degree you have received [18]?” Physical activity was assessed by the question “Over the past 30 days, did you do any vigorous activities for at least 10 minutes that caused heavy sweating, or large increases in breathing or heart rate?” (yes/no). If watching TV or videos and using computer past 30 days ≥ 3 h, screen time was defined as yes [19]. Attention deficit disorder (ADD) was assessed by the question “Has a doctor or health professional ever told you that you had ADD?” (yes/no) [20]. Seen mental health professional was assessed by the question “During the past 12 months, have you seen or talked to a mental health professional such as a psychologist, psychiatrist, psychiatric nurse or clinical social worker about your health?” (yes/no) [21]. Mother smoked when pregnant was assessed by the question “Did the participant’s biological mother smoke at any time while she was pregnant with participant?” (yes/no) [22]. Maternal age was obtained by the question “How old was participant’s biological mother when s/he was born [23]?” BMI at or above the 85th and below the 95th sex-specific percentile of the BMI-for-age growth chart was defined as overweight according to the CDC [24]. Obesity was defined as BMI at or above the sex-specific 95th percentile of the CDC BMI-for-age growth chart. Underweight was defined as having BMI at or above the 5th percentile and normal weight was defined as having a BMI at or above the 5th percentile and below the 85th percentile, for age and gender [25]. ADD drug was identified based on participants’ self-reported use of the following drugs: central nervous system stimulants, methylphenidate and atomoxetine.

### Statistical analysis

All statistical analyses were performed by *R* version 4.3.1 (2023-06-16 ucrt) and final sample was weighted with SDMVPSU, SDMVSTRA and WTMEC4YR (1999–2002) or WTMEC2YR (2003–2004). SDMVPSU is the masked variance unit pseudo-substrate is sdmvstra, and the masked variance unit pseudo-primary sampling units was sdmvpsu. SDMVSTRA refers to the CI being applied to assess the reliability of an estimate. WTMEC4YR and WTMEC2YR are the mobile examination center (MEC) exam weight used for weighting.

Continuous data were expressed as mean and standard error (S.E.), and the weighted t-test was applied for the comparison between two groups. Categorical data were described by the number of cases and percentage [*N* (%)], and the Rao-Scott chi-square test was used for comparison between LD and non-LD group. The weighted univariable and multivariable logistic regression models were used to evaluate the relationship of tobacco smoke exposure and DHA intake on childhood LD, with odds ratios (ORs) and 95% confidence intervals (CIs). Importantly, we assessed the joint effect of DHA intake and tobacco smoke exposure on LD. In multivariate logistic regressions, model 1 was a crude model, and model 2 adjusted age, gender, PIR, attention deficiency, seen mental health professional, mother smoked when pregnant and BMI. Subgroup analysis was performed in different age, gender, BMI, whether attention deficiency or seen mental health professional groups. The trend of joint effect of DHA intake and smoke exposure of LD in children and adolescents were analyzed using Mann-Kendall trend test. Two-sided *P* < 0.05 was considered to reflect a statistically significant difference.

## Results

### Children’s characteristics

A total of 5,247 eligible children and adolescents were finally included, with the mean age of 10.67 (0.05) years. In this population, the odds of LD in children and adolescents was 11.30%. The characteristics of the study children and adolescents were listed in Table 1. Children and adolescents with non-LD had a higher rate of DHA intake than those with LD (64.60% vs. 57.21%, *P* = 0.06), while the exposure rate of smoking in LD group was higher than those without LD (75.06% vs. 56.46%, *P* < 0.01). Differences were found in age, gender, race, the level of PIR, household education, birth weight and BMI, maternal age, with or without the history of attention deficiency, seen mental health professional, maternal smoking during pregnancy, the use of psychotherapeutic drug, ADHD drug, DHA intake and smoking exposure between the two groups (*P* < 0.05).

**Table 1:** Characteristic of children

Variables	Total (*n* = 5247)	Non-LD (*n* = 4654)	LD (*n* = 593)	Statistics	*P*
Age, years, Mean (± S.E)	10.67 (± 0.05)	10.59 (± 0.04)	11.26 (± 0.20)	t = 3.218	0.002
Gender, *n* (%)				χ² = 18.156	< 0.001
Female	2660 (48.56)	2433 (50.21)	227 (35.76)		
Male	2587 (51.44)	2221 (49.79)	366 (64.24)		
Race, *n* (%)				χ² = 4.104	0.022
Black	1662 (14.93)	1439 (14.58)	223 (17.58)		
Others	2226 (24.54)	2027 (25.24)	199 (19.11)		
White	1359 (60.53)	1188 (60.18)	171 (63.30)		
PIR, *n* (%)				χ² = 7.961	< 0.001
< 1	1626 (21.97)	1383 (20.51)	243 (33.27)		
> 3.5	978 (27.64)	909 (29.00)	69 (17.10)		
1-1.85	1229 (21.69)	1099 (21.46)	130 (23.50)		
1.86–3.5	1082 (23.98)	961 (24.29)	121 (21.50)		
Unknown	332 (4.72)	302 (4.73)	30 (4.64)		
Health insurance, *n* (%)				χ² = 3.371	0.051
No	902 (13.45)	817 (13.23)	85 (15.18)		
Unknown	56 (0.67)	48 (0.56)	8 (1.55)		
Yes	4289 (85.88)	3789 (86.21)	500 (83.27)		
Household education, *n* (%)				χ² = 4.400	0.010
Above high school	1894 (47.97)	1724 (49.26)	170 (37.95)		
Below high school	749 (7.73)	673 (7.61)	76 (8.63)		
High school	2461 (41.89)	2130 (40.71)	331 (50.99)		
Unknown	143 (2.41)	127 (2.41)	16 (2.43)		
Physical activity, met·min, *n* (%)				χ² = 3.943	0.053
No	2693 (61.01)	2408 (61.70)	285 (55.67)		
Yes	2554 (38.99)	2246 (38.30)	308 (44.33)		
Screen time, *n* (%)				χ² = 3.736	0.060
No	3650 (70.67)	3247 (71.16)	403 (66.90)		
Yes	1597 (29.33)	1407 (28.84)	190 (33.10)		
Attention deficit disorder, *n* (%)				χ² = 240.452	< 0.001
No	4863 (90.86)	4461 (94.55)	402 (62.28)		
Yes	384 (9.14)	193 (5.45)	191 (37.72)		
Seen mental health professional, *n* (%)				χ² = 76.352	< 0.001
No	4798 (89.65)	4374 (92.37)	424 (68.53)		
Yes	449 (10.35)	280 (7.63)	169 (31.47)		
Day care or preschool, *n* (%)				χ² = 0.152	0.698
No	1718 (27.07)	1539 (26.98)	179 (27.78)		
Yes	3529 (72.93)	3115 (73.02)	414 (72.22)		
Birth weight, pounds, *n* (%)				χ² = 10.019	0.003
< 5.5	630 (10.66)	529 (10.00)	101 (15.76)		
≥ 5.5	4617 (89.34)	4125 (90.00)	492 (84.24)		
Maternal smoking during pregnancy, *n* (%)				χ² = 43.642	< 0.001
No	4480 (81.28)	4042 (83.19)	438 (66.50)		
Yes	767 (18.72)	612 (16.81)	155 (33.50)		
Maternal age, years, Mean (± S.E)	26.26 (± 0.20)	26.38 (± 0.20)	25.33 (± 0.37)	t = -3.168	0.003
BMI, kg/m^2^, *n* (%)				χ² = 5.816	0.001
Normal	2995 (59.40)	2690 (60.39)	305 (51.75)		
Underweight	108 (2.82)	86 (2.43)	22 (5.83)		
Overweight	966 (18.10)	862 (18.28)	104 (16.71)		
Obesity	1178 (19.68)	1016 (18.91)	162 (25.72)		
Energy, kcal, Mean (± S.E)	2108.35 (± 22.96)	2102.93 (± 23.11)	2150.43 (± 65.57)	t = 0.724	0.473
WBC, 1000 cells/uL, *n* (%)				χ² = 0.059	0.888
< 6.8	2691 (48.67)	2372 (48.74)	319 (48.10)		
≥ 6.8	2555 (51.32)	2281 (51.25)	274 (51.90)		
Unknown	1 (0.01)	1 (0.01)	0 (0.00)		
Psychotherapeutic drug, *n* (%)				χ² = 25.529	< 0.001
No	5163 (97.71)	4606 (98.47)	557 (91.79)		
Yes	84 (2.29)	48 (1.53)	36 (8.21)		
ADHD drug, *n* (%)				χ² = 82.409	< 0.001
No	5074 (95.25)	4554 (96.88)	520 (82.66)		
Yes	173 (4.75)	100 (3.12)	73 (17.34)		
DHA intake, *n* (%)				χ² = 8.331	0.006
No	1866 (36.24)	1632 (35.40)	234 (42.79)		
Yes	3381 (63.76)	3022 (64.60)	359 (57.21)		
Tobacco smoke exposure, *n* (%)				χ² = 29.056	< 0.001
No	2176 (41.41)	2031 (43.54)	145 (24.94)		
Yes	3071 (58.59)	2623 (56.46)	448 (75.06)		

χ^2^: chi-square test; t: t-test; S.E: standard error

LD: learning disabilities; PIR: poverty income ratio; PIR < 1: poor; PIR 1-1.85: near poor; PIR: 1.86–3.5, middle income; PIR > 3.5: higher income; BMI: body mass index; met: metabolic equivalent; WBC: white blood cell; ADHD: attention deficit hyperactivity disorder; DHA: docosahexaenoic acid

### Association of DHA intake and tobacco smoke exposure and their joint effect on

#### LD in children and adolescents

Table 2 shown the relationship of DHA intake and tobacco smoke exposure and childhood LD, respectively. In model 1, comparison to the non-DHA intake group, the OR (95%CI) of LD in DHA intake group was 0.73 (0.59–0.91) (*P* = 0.006). In model 2, after adjusting age, gender, PIR, attention deficiency, seen mental health professional, maternal smoking during pregnancy and BMI, DHA intake group was also related to the lower incidence of LD (OR = 0.76, 95%CI: 0.61–0.96, *P* = 0.028).

**Table 2:** Joint effect between DHA intake and tobacco smoke exposure on children’s LD

Variables	Model 1		Model 2	
	OR (95% CI)	*P*	OR (95% CI)	*P*
DHA intake				
No	Ref		Ref	
Yes	0.73 (0.59–0.91)	0.006	0.76 (0.61–0.96)	0.028
Tobacco smoke exposure				
No	Ref		Ref	
Yes	2.32 (1.70–3.17)	< 0.001	1.54 (1.07–2.23)	0.028
DHA intake vs. Tobacco smoke exposure				
Yes-No	Ref		Ref	
No-No	1.86 (1.24–2.81)	0.005	1.78 (1.09–2.90)	0.028
Yes-Yes	2.79 (1.99–3.92)	< 0.001	1.82 (1.16–2.85)	0.014
No-Yes	3.18 (2.24–4.51)	< 0.001	2.08 (1.37–3.17)	0.002
Trend for Statistics	Z = 2.0381			
Trend for *P*	*P* = 0.042			

Ref: reference; OR: odds ratio; CI: confidence interval;

LD: learning disabilities. DHA: docosahexaenoic acid.;

Model 1: crude model;

Model 2: adjusted age, gender, PIR, attention deficit disorder, seen mental health professional, maternal smoking during pregnancy and BMI

The ORs with 95% CIs of LD in children and adolescents according to whether exposed to smoke were also shown in Table 2. Compared to non-tobacco smoke exposure group, tobacco smoke exposure group has a crude OR (95%CI) of 2.32 (1.70–3.17) (*P* < 0.01) for LD. After adjusted all covariates in model 2, tobacco smoke exposure was also associated with the high odds of LD (OR = 1.54, 95%CI: 1.07–2.23, *P* = 0.028).

Next, we divided the whole population into four groups according to DHA intake and whether they were tobacco smoke exposure or not (group 1: DHA intake and non-tobacco smoke exposure; group 2: non-DHA intake and non-tobacco smoke exposure; group 3: DHA intake and tobacco smoke exposure; group 4: non-DHA intake and tobacco smoke exposure). As shown in Table 2, group 1 was set as the reference, after adjusting the confounding factors, the multivariate-adjusted ORs with 95%CI of LD in children for other three groups in order were 1.78 (1.09–2.90), 1.82 (1.16–2.85) and 2.08 (1.37–3.17), respectively.

#### Joint effect of tobacco smoke exposure and DHA intake based on different age, gender, BMI, with or without attention deficit disorder and seen mental health professional

**Fig. 2:**
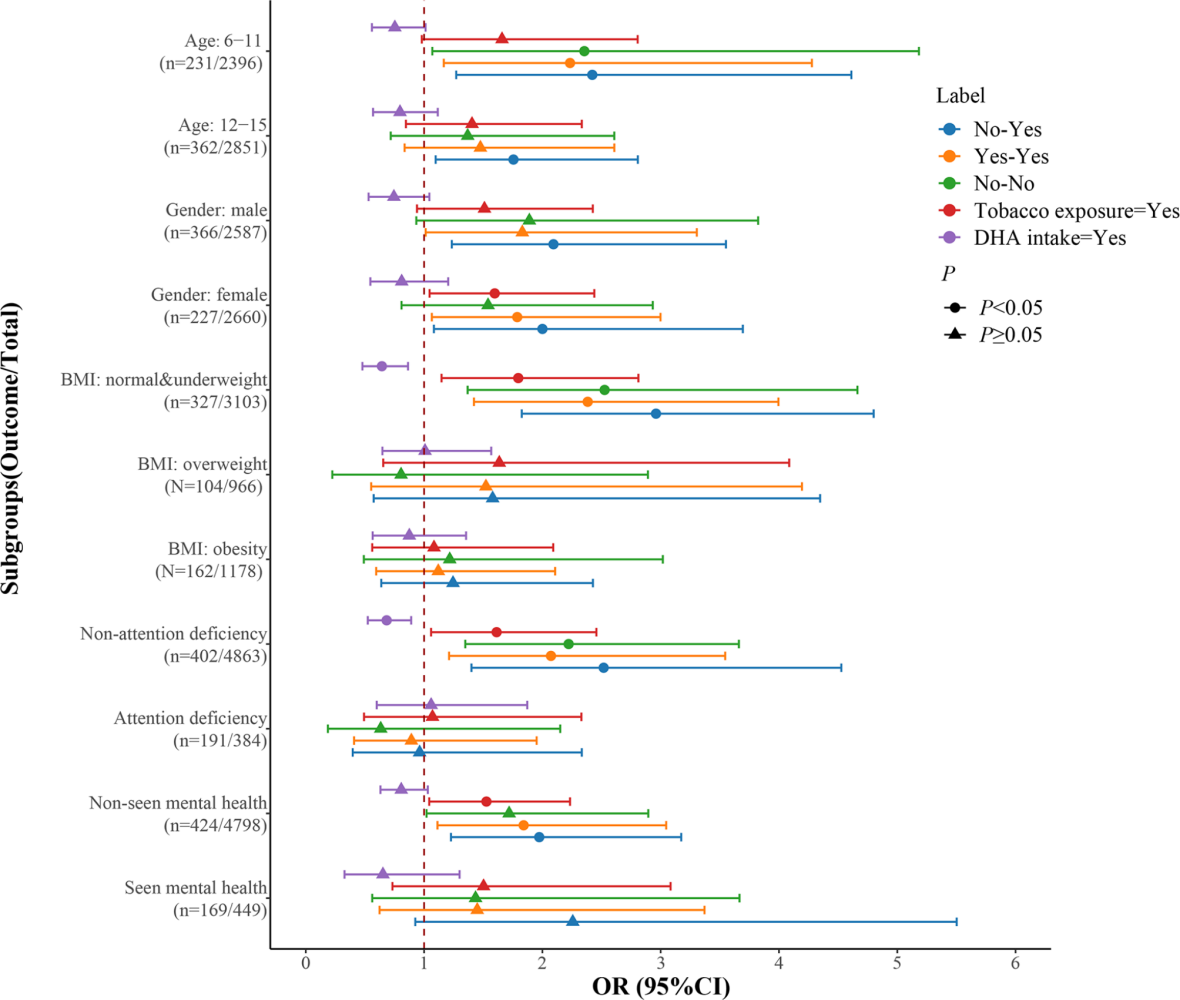
Joint effect of tobacco smoke exposure and DHA intake stratified by age, gender, BMI, with or without attention deficit disorder and seen mental health professional

Table 3; Fig. 2 shown the joint effect of tobacco smoke exposure and DHA intake based on age (6–11, 12–15 years old), gender (male, female), BMI (normal & underweight, overweight and obesity), attention deficiency (yes, no) and seen mental health professional (yes, no). After adjusting confounding factors, tobacco smoke exposure was related to the high odds of LD in female children aged 6–15 years with normal & underweight level of BMI, without the history of attention deficiency and seen mental health professional. DHA intake was related to the low odds of LD in children and adolescents aged 6–15 years with normal & underweight level of BMI and without attention deficit disorder. Non-DHA intake and tobacco smoke exposure were associated with the high odds of LD, especially among children and adolescents with normal & underweight level of BMI, without the history of attention deficit disorder and seen mental health professional.

**Table 3:** Joint effect between DHA intake and tobacco smoke exposure on children’s LD based on age, gender, BMI, the history of ADD and seen mental health professional

Subgroups (No.LD/Total)	OR (95% CI)	*P*	*N*(%)
6–11 years (*No.*231/2396)			
DHA intake			
No	Ref		773(33.83)
Yes	0.75 (0.56–1.01)	0.070	1623(66.17)
Tobacco smoke exposure			
No	Ref		969(40.59)
Yes	1.66 (0.98–2.81)	0.068	1427(59.41)
DHA intake vs. Tobacco smoke exposure			
Yes-No	Ref		674(28.85)
No-No	2.35 (1.07–5.18)	0.042	295(11.74)
Yes-Yes	2.23 (1.17–4.28)	0.022	949(37.32)
No-Yes	2.42 (1.27–4.61)	0.012	478(22.09)
12–15 years (*No*.362/2851)			
DHA intake			
No	Ref		1093(39.43)
Yes	0.80 (0.57–1.12)	0.195	1758(60.57)
Tobacco smoke exposure			
No	Ref		1207(42.51)
Yes	1.41 (0.85–2.33)	0.198	1644(57.49)
DHA intake vs. Tobacco smoke exposure			
Yes-No	Ref		748(27.24)
No-No	1.37 (0.72–2.61)	0.348	459(15.27)
Yes-Yes	1.48 (0.84–2.61)	0.190	1010(33.33)
No-Yes	1.76 (1.10–2.81)	0.026	634(24.16)
Female (*No*.227/2660)			
DHA intake			
No	Ref		924(34.97)
Yes	0.81 (0.55–1.20)	0.306	1736(65.03)
Tobacco smoke exposure			
No	Ref		1112(41.31)
Yes	1.60 (1.05–2.44)	0.038	1548(58.69)
DHA intake vs. Tobacco smoke exposure			
Yes-No	Ref		744(28.80)
No-No	1.54 (0.81–2.93)	0.198	368(12.51)
Yes-Yes	1.79 (1.07-3.00)	0.036	992(36.23)
No-Yes	2.00 (1.08–3.69)	0.035	556(22.46)
Male (*No*.366/2587)			
DHA intake			
No	Ref		942(37.44)
Yes	0.74 (0.53–1.04)	0.097	1645(62.56)
Tobacco smoke exposure			
No	Ref		1064(41.51)
Yes	1.51 (0.94–2.43)	0.098	1523(58.49)
DHA intake vs. Tobacco smoke exposure			
Yes-No	Ref		678(27.55)
No-No	1.89 (0.93–3.82)	0.087	386(13.97)
Yes-Yes	1.83 (1.01–3.31)	0.054	967(35.01)
No-Yes	2.09 (1.23–3.55)	0.010	556(23.47)
Non-ADD (*No*.402/4863)			
DHA intake			
No	Ref		1729(36.07)
Yes	0.68 (0.52–0.89)	0.008	3134(63.93)
Tobacco smoke exposure			
No	Ref		2079(42.79)
Yes	1.61 (1.06–2.46)	0.033	2784(57.21)
DHA intake vs. Tobacco smoke exposure			
Yes-No	Ref		1354(29.18)
No-No	2.22 (1.35–3.66)	0.004	725(13.60)
Yes-Yes	2.07 (1.21–3.55)	0.012	1780(34.74)
No-Yes	2.52 (1.40–4.53)	0.004	1004(22.47)
ADD (*No*.191/384)			
DHA intake			
No	Ref		137(37.91)
Yes	1.06 (0.60–1.87)	0.843	247(62.09)
Tobacco smoke exposure			
No	Ref		97(27.74)
Yes	1.07 (0.49–2.33)	0.864	287(72.26)
DHA intake vs. Tobacco smoke exposure			
Yes-No	Ref		68(17.90)
No-No	0.63 (0.19–2.15)	0.471	29(9.84)
Yes-Yes	0.89 (0.41–1.95)	0.778	179(44.19)
No-Yes	0.96 (0.40–2.33)	0.933	108(28.07)
Non-seen mental health professional *(No*.424/4798)			
DHA intake			
No	Ref		1714(36.33)
Yes	0.81 (0.63–1.03)	0.097	3084(63.67)
Tobacco smoke exposure			
No	Ref		2045(42.86)
Yes	1.53 (1.04–2.23)	0.037	2753(57.14)
DHA intake vs. Tobacco smoke exposure			
Yes-No	Ref		1334(29.14)
No-No	1.72 (1.02–2.90)	0.051	711(13.72)
Yes-Yes	1.84 (1.11–3.05)	0.024	1750(34.53)
No-Yes	1.97 (1.23–3.17)	0.009	1003(22.61)
Seen mental health professional (*No*.169/449)			
DHA intake			
No	Ref		152(35.48)
Yes	0.65 (0.33–1.30)	0.234	297(64.52)
Tobacco smoke exposure			
No	Ref		131(28.92)
Yes	1.50 (0.73–3.08)	0.276	318(71.08)
DHA intake vs. Tobacco smoke exposure			
Yes-No	Ref		88(19.63)
No-No	1.43 (0.56–3.67)	0.457	43(9.29)
Yes-Yes	1.45 (0.62–3.37)	0.396	209(44.89)
No-Yes	2.26 (0.93–5.50)	0.085	109(26.20)
BMI: Normal & Underweight (*No*.327/3103)			
DHA intake			
No	Ref		1099(36.67)
Yes	0.64 (0.48–0.86)	0.006	2004(63.33)
Tobacco smoke exposure			
No	Ref		1346(44.43)
Yes	1.80 (1.15–2.81)	0.015	1757(55.57)
DHA intake vs. Tobacco smoke exposure			
Yes-No	Ref		875(30.07)
No-No	2.53 (1.37–4.66)	0.006	471(14.37)
Yes-Yes	2.38 (1.42-4.00)	0.002	1129(33.26)
No-Yes	2.96 (1.83–4.80)	< 0.001	628(22.30)
BMI: Overweight (*No*.104/966)			
DHA intake			
No	Ref		320(31.34)
Yes	1.01 (0.65–1.57)	0.973	646(68.66)
Tobacco smoke exposure			
No	Ref		391(40.79)
Yes	1.64 (0.65–4.09)	0.300	575(59.21)
DHA intake vs. Tobacco smoke exposure			
Yes-No	Ref		268(29.66)
No-No	0.81 (0.22–2.89)	0.742	123(11.12)
Yes-Yes	1.52 (0.55–4.19)	0.422	378(39.00)
No-Yes	1.58 (0.57–4.35)	0.383	197(20.22)
BMI: Obesity (*No*.162/1178)			
DHA intake			
No	Ref		447(39.39)
Yes	0.88 (0.57–1.35)	0.554	731(60.61)
Tobacco smoke exposure			
No	Ref		439(32.45)
Yes	1.08 (0.56–2.09)	0.812	739(67.55)
DHA intake vs. Tobacco smoke exposure			
Yes-No	Ref		279(20.72)
No-No	1.22 (0.49–3.02)	0.675	160(11.73)
Yes-Yes	1.12 (0.60–2.11)	0.727	452(39.88)
No-Yes	1.24 (0.64–2.43)	0.526	287(27.67)

Ref: reference; OR: odds ratio; CI: confidence interval;

LD: learning disabilities. DHA: docosahexaenoic acid; ADD: attention deficit disorder

## Discussion

In present study, we found there was a joint effect between DHA intake and tobacco smoke exposure on the odds of LD in American children and adolescents. This joint effect remained robust when subgroup analyses were performed. Ensuring DHA intake while reducing tobacco smoke exposure may have potential benefits in the preventing of LD in children.

Previous studies have supported the association of tobacco smoke exposure and a high risk of children’s LD. A NHANES suggested that tobacco smoke exposure was related to increased risk of LD in children after adjusting age, race, gender, PIR, mother smoked while pregnant, smoker at home, birth weight and blood lead [26]. Research of Kibir et al. [27] reported approximately 4.8 million U.S. children aged ≤ 12 years old are exposed to second hand smoking in their homes, and 3–8% suffer from 1 or more neurobehavioral disorders. Another large sample study also from the NSCH found tobacco smoke exposure was associated with several mental health and neurodevelopment. Children who lived with smoker have an increased risk of mental health problems such as LD and attention deficit disorders [28]. Our results were in line with above researches, the odds of LD were increased by 54% in children exposed to tobacco smoke compared with children non-exposure to tobacco smoke. Children were reported to be more sensitive to tobacco smoke exposure than adults due to their special metabolic characteristics [29, 30]. From the mechanisms level, both oxidative stress, which results from oxidative imbalance by secondary activation of intrinsic sources of reactive oxygen species (ROS), and inflammation are responsible for most of negative effects of smoking [10]. The ability of tobacco smoke to cause oxidative stress and systemic inflammation has far-reaching consequences. A population-based cohort study lasted 30 years showed that compared to non-exposed, individual exposed to non-hygienic parental smoking was at higher risk for poor midlife episodic memory and associative learning [31]. Controlling tobacco use and reducing exposure to tobacco smoke may have a long-term benefit for children’s physical and mental development.

DHA is one of the most important marine n-3 PUFA in the human diet and has a strong effect on brain function and structure of the neuronal cell membranes. Long-term DHA deficiency may increase the incidence of mood and behavior disorders in children [32]. One randomized controlled trial (RCT) in south African children with low fish intake reported a beneficial effects of a fortified with fish flour on verbal recognition and spelling [33]. Another study focused on UK children also suggested the influence of a three-month intervention with DHA (174 mg/d) on reading age and working memory but not on behavior [34]. Our results were consistent with these findings. However, there were still inconsistencies in the reported literature. Several studies shown that no relationship between DHA intake and a beneficial effect on cognitive outcomes in children aged 4–14 years old. Kennedy et al. [35] reported that no beneficial effects of DHA supplementation for 8 weeks on brain function were observed in cognitively intact children. Moreover, another study indicated that DHA-rich supplementation during pregnancy did not improve cognitive and language development in early childhood [36]. Further research is worth being performed on the hypothesis that DHA intake decreases the risk of LD in childhood.

Our results indicated there was a joint effect between DHA intake and smoking exposure on LD in children and adolescents. Compared with DHA intake and non-smoking exposure, children and adolescents with non-DHA and exposed to smoke had a significant higher incidence of LD. Tobacco smoke and its oxygen radicals activate inflammatory cells, which contribute to the imbalance of protease-antiprotease and advancing tissue destruction. DHA has been shown to reduce oxidative stress caused by active smoking and improve vascular function in both in human subjects and experimental animal models [37–39]. Previous studies have shown that active smokers have significantly lower levels of DHA, compared to nonsmokers. Simon et al. [40] suggested smoking and alcohol consumption may have an influence on absorption, synthesis, or metabolism of serum fatty acids. Moore et al. [12] shown that joint effect between high exposure to second hand smoking and low omega-3 PUFA intake level on metabolic syndrome risk among children was stronger than would be expected from the effects of the single exposure factor. In subgroup analyses, tobacco smoke exposed children in all age groups were at high odds of LD. Snice virtually all cigarette smoking begins before the age of 18, the effects of tobacco use on children was clearly of utmost importance. The subgroup results of our study also showed a higher odds of LD among girls exposed to tobacco smoke. Previous studies have demonstrated that smoking appears to be more harmful for female compared with male [41], although these studies have focused more on female in the childbearing age group. Active or passive smoking may cause menstrual disorders, low estrogen and other adverse consequences.

Our study has several strengths. Firstly, to our knowledge, this was the first research to evaluate the association between the DHA intake and tobacco smoking exposure on the odds of children and adolescents’ LD, which has significant public health relevance. Our study provides theoretical support for the importance of DHA intake while reducing the tobacco smoke exposure. There was a greater need for children and adolescents exposed to tobacco to consume DHA-rich food and supplements. Secondly, a representative NHANES database was used, and the regression models were adjusted for relevant potential variables to make the results credible and robust. There were still several limitations in our study. Firstly, due to the cross-sectional design, it was difficult to determine the causality. Secondly, DHA intake data were obtained from FFQ and may have recall bias. Moreover, in the multivariate logistic regression model, as many covariates related to LD as possible were included, but the confounding effect of missing or unknown factors still cannot be excluded.

## Conclusion

In summary, there was a joint effect of DHA intake and tobacco smoke exposure on LD among children and adolescents. These results could help develop more guiding targeted strategies, including a complete ban or stricter restrictions on smoking, and the use of DHA supplements to prevent and control children and adolescents’ LD around the world. Indeed, future prospective studies are needed for evaluating the impact of tobacco smoke exposure and DHA intake on the children and adolescents with LD.

## Electronic supplementary material

Below is the link to the electronic supplementary material.

Supplementary Material 1

## Data Availability

Ethical approval was not provided for this study on human participants because NHANES is a publicly available dataset, this data can be found here NHANES, *NHANES Questionnaires*,* Datasets*,* and Related Documentation (cdc.gov)*.

## Abbreviations

LD: Learning disabilities
DHA: Docosahexaenoic acid
PUFA: Polyunsaturated fatty acid
ADHD: Attention deficit hyperactivity disorder
NHANES: National Health and Nutrition Examination Surveys
NCHS: National Centers for Health Statistics
CDC: Centers for Disease Control and Prevention
BMI: Body mass index
FFQ: Food frequency questionnaire
PIR: Poverty-to-income ratio
MEC: Mobile examination center
ORs: Odds ratios
CIs: Confidence intervals
RCT: Randomized controlled trial
ROS: Reactive oxygen species
