# Supplementary material for: Joint effect of docosahexaenoic acid intake and tobacco smoke exposure on learning disability in children and adolescents: a cross-sectional study from the NHANES database
Source: Ital J Pediatr. 2024 Sep 27;50:197. doi: 10.1186/s13052-024-01745-3 (PMC11438000; doi:10.1186/s13052-024-01745-3)
Supplement: Supplementary file 1 — Supplementary Material 1 [file 13052_2024_1745_MOESM1_ESM.docx]

Reviewer reports:

Reviewer #1: The authors have done a good job of editing and revising the manuscript, I just have some typos and suggestions regarding journal style:

Q1: Page 8 line 17 (Dowling et...) Please add reference to journal style.

Reply: Thank you for your comment. We have revised this reference style.

Q2: Page 15 Line 51 "from 1or..." let's add a space in between.

Reply: Thank you for your comment. We have added a space in 1 and or.

Q3: Include the abbreviations (ROS) you used in the revision in "abbreviations".

Reply: Thank you for your comment. We have added the ROS in the “abbreviations”.

Q4: Give doi numbers in your references as "https://doi.org/.... " in your references.

Reply: Thank you for your comment. We have added the DOI in the references.

Q5: Please proofread the article for minor spelling and grammatical mistakes.

Reply: Thank you for your comment. We have checked the whole article carefully and corrected the grammar and spelling errors.

Reviewer #2: Dear author,

You had better highlight only the sentences that need revision, not the general part.

You didn't change some sentences, but you highlighted them.

I felt its somewhat was close to cheating.

Meanwhile, I accept your correction.

Reply: Thank you for your comment and accepting our corrections. Based on your comment, I will only highlight the changes in the revision in the future to save ourselves from suspicion of cheating.
